# The metaphysical underdetermination of time-reversal invariance

**DOI:** 10.1007/s11229-023-04039-z

**Published:** 2023-01-12

**Authors:** Cristian López

**Affiliations:** University of Lausanne, Lausanne, Switzerland

**Keywords:** Time reversal, Direction of time, Time, Laws, Quantum mechanics, Classical mechanics, Classical electromagnetism

## Abstract

In this paper I argue that the concept of time-reversal invariance in physics suffers from metaphysical underdetermination, that is, that the concept may be understood differently depending on one’s metaphysics about time, laws, and a theory’s basic properties. This metaphysical under-determinacy also affects subsidiary debates in philosophy of physics that rely on the concept of time-reversal invariance, paradigmatically the problem of the arrow of time. I bring up three cases that, I believe, fairly illustrate my point. I conclude, on the one hand, that any formal representation of time reversal should be explicit about the metaphysical assumptions of the concept that it intends to represent; on the other, that philosophical arguments that rely on time reversal to argue against a direction of time require additional premises.

## Introduction

There has lately been some interest in the philosophy of physics community around the concept of ‘time-reversal invariance’. On the one hand, some discussions centered in which is the right implementation of time reversal in different physical theories, mainly in classical electromagnetism and non-relativistic quantum mechanics (see, for instance, Savitt, 1996; Callender, 2000; Albert, 2000; Earman, 2002; Malament, 2004; North, 2008; Arntzenius & Greaves, 2009; Peterson, 2015; Roberts, 2017; Allori, 2019; Lopez, 2021; Struyve, 2022 among others). On the other, some have disputed, or defended, the philosophical relevance of time-reversal *invariance* in relation to the problem of the direction of time (Sklar, 1974; Earman, 1974; Horwich, 1987; Callender, 1995; Hutchison, 1993, 1995; Savitt, 1996; Arntzenius, 1997; Lopez & Esfeld, 2022). Although they are frequently discussed separately, a relation exists. For the one thing, that a physical theory be time-reversal invariant would in principle and with some qualifications shed some light on the temporal structure of the theory (e.g., whether it comes equipped with a direction of time). For the other, in order to know whether time-reversal invariance is instantiated in a physical theory, one needs to know whether time reversal is being implemented fairly. Discrepancies on the latter may lead to discrepancies on the former.

The aim of this paper is to show that discrepancies are deeper than usually thought. My thesis is that the concept of time-reversal invariance suffers from acute metaphysical underdetermination. That is, our *conceptual* understanding of time reversal, and consequently its formal implementation within a theoretical context, depends to a large extent on one’s metaphysical assumptions. Two lessons can be drawn from this. First, there is no univocal way to conceptually understand time-reversal invariance, but it is metaphysics-laden. Although it is not an empirical problem and nothing in the physics is to be altered, this case of underdetermination could in fact affect our philosophical understanding of the concept. Second, some philosophical problems and arguments that draw on the concept of time-reversal invariance could need some qualification or adjustment in the light of such underdetermination, such as the problem of the direction of time.

In particular, I argue that the metaphysical underdetermination of time-reversal invariance manifests itself in at least three cases. First, there is metaphysical underdetermination in non-relativistic quantum mechanics, since the concept, and thereby the formal implementation, of time reversal depends on our metaphysics of time (i.e., either relational or substantival). Second, there is metaphysical underdetermination of time reversal in classical electromagnetism, since the definition of time reversal ultimately relies on which are the basic properties of the theory and whether a temporal orientation exists independently from the series of instantaneous states. Third and finally, there is metaphysical underdetermination in classical mechanics, since the time-reversal invariance of the theory depends on adopting an ontology of forces that are dependent only on interparticle distances, setting aside non-conservative or phenomenal forces as unreal. I do not mean that all these cases of metaphysical underdetermination necessarily lead to relativism and indifference. We may have very good reasons to uphold some of the options in play, but arguments for them do not come from the physics of time reversal. The understanding and the implementation of time-reversal invariance resort to philosophical assumptions that have to be made explicit, that call for autonomous philosophical argumentation, and that can of course be challenged.

From a general perspective, this paper aims to contribute to the rich recent literature on time reversal and its relation to ontology and cases of underdeterminacy (for instance, Allori, 2019; Farr, 2020; Lopez, 2021; Porter Williams, 2022; Struyve, 2022). In some sense, my paper draws on the literature, but it seeks to make a more general, and maybe more radical, point. The underdeterminacy I want to stress is that of the concept of time reversal itself. I do this by showing different cases in which the very representation of time reversal (i.e., the series of transformations that the time-reversal transformation is supposed to carry out) crucially relies on our views on time and on the ontology of physical theories. This observation implies that the connection between time-reversal invariance and the direction of time must be revised, and I provide some guidelines on how to do so. The interest of the paper is hence twofold—it shows the metaphysical underdeterminacy of time-reversal invariance and the consequences of it for the debate about the direction of time.

The structure of the paper is as follows. In Sect. 1, I introduce the notion of time-reversal invariance distinguishing between the concept and its formal representation. I also introduce a popular argument in the literature against a primitive direction of time, which is based on time-reversal invariance. From Sects. 2 to 4, I present three cases in which the metaphysical underdetermination of time-reversal invariance emerges in different domains. In Sect. 2, in non-relativistic quantum mechanics. In Sect. 3, in classical electromagnetism. And in Sect. 4, in classical mechanics. In Sect. 5, I revisit the argument introduced in Sect. 1 as conclusion.

## Time-reversal invariance and the dynamical argument

What is time reversal and time-reversal invariance? The concept of time-reversal invariance is not easy to spell out with precision. One of the take-home messages of this paper is that there is no univocal concept of time reversal, but it depends on some metaphysical commitments previously adopted. But, before getting at this, it is worthy to show why this is important for philosophical debates. To begin, I believe that one of the most common misunderstandings in philosophy of physics about the *concept* of time reversal is that it seems to be defined by its formal implementations. This, I submit, erroneously reverses the relation between the concept and the *formal representation* of the concept. Many of the quarrels around time reversal arise because people diverge on the concept, not because they are sloppy in their representation. It is true that there seems to be a folk picture of what time reversal is, didactically illustrated by, for instance, a movie being played back and forth. It is also true, nonetheless, that this illustration may be misleading in many cases and is of limited application, if any. So, it is important to distinguish between the concept of time reversal, on the one side, and the piece of mathematics that represents it, on the other.

Second, time reversal has not only been of philosophers’ curiosity for its meaning in physics, but also for its role in philosophy. For instance, time reversal generally acts upon dynamical laws, which would suggest that divergent views of laws can lead to different concepts of time reversal (or different consequences that can follow from it). Another example is that time reversal is frequently claimed to be a symmetry, at least of some fundamental laws. But there has been some debate about what the metaphysics of symmetries is (e.g., whether they are real and fundamental, or epistemic). Thus, our philosophical understanding of time reversal could also be affected by such a debate. Nonetheless, the concept of time reversal has been particularly crucial in debates around the direction of time or causation. Though I will center the discussion in the connection with the direction of time, I will also refer to the relation between time reversal and laws, time reversal and causation, time reversal and symmetries, and time reversal and a theory’s ontology to show how metaphysical underdetermination arises in different cases.

Since the late nineteenth-century, whether the dynamical equations of a physical theory turn out time-reversal invariant or not was supposed to be crucial for problems gravitating around irreversible processes and the direction of time (see Boltzmann, 1872; Loschdmidt 1876; Reichenbach, 1956, among many others). The belief was (and to some extent still is) that time-reversal invariant laws speak *against* a primitive (or fundamental) direction of time in physics^1^ (see Mehlberg, 1961; Horwich, 1987; Price, 1996; Artntzenius, 1997; Maudlin, 2002; Loewer, 2012). The argument, which I call “the dynamical argument against a primitive arrow of time” (the “Dynamical Argument”, for short) can be sketched as follows:

P1. If the dynamics of our best physical theories is time-reversal invariant, then a primitive direction of time is unwarranted.^2^

P2. It happens that our best physical theories are time-reversal invariant

C. Therefore, a primitive direction of time is unwarranted.

Some points of the argument are noteworthy. To begin, it does not imply that a direction of time is just a mere illusion. There may be other non-dynamical ways to show that the direction of time is not an illusion (e.g., special boundary conditions). The argument allegedly shows that if there is a direction of time, it is not *built into* the dynamics of physical theories, but that a reductive, non-primitivist approach should be rather adopted. Second, a similar argument has been employed to make a case against causation (see Russel, 1913). Indeed, the relation between time-reversal invariance and causation has drawn some interest lately (Price and Weslake 2010; Farr, 2020; Porter Williams, 2022). Some have thought that time-reversed worlds have their causal relations reversed. Others thought that such an interpretation is a mistake. For instance, Porter Williams (2022) argues that time-reversed worlds lack causal relations at all. The argument is that if causation is a difference-making relation, the inversion of the temporal ordering cannot result in a world with causal relations reversed, but in a world without causal relations (2022, p. 94). Even though I am sympathetic with Porter William’s rationale, I will not dig into the relation between time-reversal invariance and causation, but into the metaphysical assumptions that guide physically and formally alternative ways to represent the action of reversing the direction of time.

Time reversal has thereby a twofold value—in itself and in its role in the Dynamical Argument. Naturally, if the concept of time reversal is shown to be metaphysically underdetermined, it will affect the completeness of the argument as it stands. That is, if time reversal can be conceptualized in different ways and this has consequences for its implementation, then the argument, to work as intended, would need to be furnished with additional premises that capture the additional content. This is in fact my second thesis in the article: if the concept of time reversal is metaphysically underdetermined, arguments based on time reversal require additional premises to work successfully in a philosophical context. One of the corollaries of this is that the Dynamical Argument cannot prove that there is not primitive direction of time of time if not supplied with additional premises.

I have said before that it is important to be clear about the relation between the concept of time reversal and its formal implementations. It is worth emphasizing that the implementation of time reversal is theory relative. This means that the formal features of the mathematical transformation that implements the concept of time reversal vary across theories. This does not mean that the *concept* be also theory relative. It may be the case that the implementation of the same concept requires different formal resources depending on the theory at stake—how time reversal must be formally represented is to be decided within the formal structure of a specific physical theory, though the concept remains the same. Suppose that time reversal is to be conceptualized in terms of motion reversal. Though this concept is to be held across theories, the representation of motion within a dynamical structure can be different. This would ipso fact entail that the representation of time reversal as motion reversal can also be different (see Lopez, 2019).

Second, the implementation of the time-reversal transformation can prima face be regarded as active or passive. Suppose a set of classical dynamical laws *L* for a set of trajectories $q\left( t \right)$ in the coordinate system *O.* The *passive* time-reversal transformation amounts to rewriting the dynamical laws *L* for $q\left( t \right)$ in a different coordinate system, $O^{\prime}$, where $O^{\prime} = O^{T}$ is the time-reversed coordinate system. The time-reversal transformation is therefore given by $T:O \to O^{T}$, which yields $T:L_{o} \to L_{{o^{T} }}$, that is, from the laws as written in one coordinate system to the laws written in a time-reversed coordinate system. In this case, the transformation of time reversal corresponds simply to a translation into the language of $O^{T}$ of the dynamical laws written in the language of *O* for a set of trajectories. It can then be said that the time-reversal transformation yields different *descriptions* of the same trajectories. If so-considered transformation leaves invariant the mathematical form of the dynamical laws, then time-reversal *invariance* holds.

In the active view, the time reversal transformation is not a mere redescription. Even though, there would be at least two different active views (see Fonda & Ghirardi, 1970, Ch. 1.7), I assume here that the active time-reversal transformation *T* amounts to reversing the trajectories themselves, $T:q\left( t \right) \to q^{T} \left( { - t} \right)$, where $q$ is a curve on state-space parametrized by time ($t$). Thus, the time-reversal transformation yields a *new* physical evolution ($q^{T} \left( { - t} \right)$) as considered from the same coordinate system, *O*. An alternative way to consider it is by imagining that the time-reversed trajectories are solutions of a time-reversed dynamical law, $T:L \to L^{T}$. While the set *L* generates the set of trajectories $q\left( t \right)$, $L^{T}$ generates $q^{T} \left( { - t} \right)$ (see Castagnino & Lombardi, 2009). Naturally, the generation of time-reversed trajectories can imply the transformation not only of the time coordinate, but also of other dynamical magnitudes (e.g., the momenta, the velocities, etc.). Time-reversal invariance can be assessed now from the perspective of the space of solutions of the dynamical laws—time-reversal invariance holds if, if $q\left( t \right)$ is a possible solution of *L* then $q^{T} \left( { - t} \right)$ is also a possible solution of *L,* where $L$ is equivalent to $L^{T}$. In this sense, the active time-reversal transformation is said to be space-solution preserving (if time-reversal invariance holds).

In the light of the Dynamical Argument sketched above, the interesting view of time reversal is the active one. In the passive view, it is blatant that a mere *re*-description of the language in which dynamical equations are written down should not produce any physical change. For instance, the transformation of the time coordinate, $T:t \to - t$, is viewed as a mere change in the convention on the sign of the time coordinate; for consistency with the convention, we must surely change other conventions (like the sign of *p*). If the Dynamical Argument is to be an interesting philosophical argument, P2 and the antecedent of P1 cannot merely mean that the language in which we write our physical theories down allow for changes in the conventions. If the argument is meant to say something substantial about the world through the notion of time reversal, then how can a feature of the world be somehow related to our linguistic conventions?

In the active case, this worry disappears. The mathematical implementation of the transformations is meant to represent a physical change, in the sense that they are meant to formally capture not a change in linguistic conventions, but some physical transformation.^3^ Note, however, that the mathematical implementation (the representation) cannot completely define what the physical transformations is (what is represented). It is rather the other way around—some intuitions about, and conditions for, the physical transformation guide its formal implementation. That’s why it is helpful to be clear about the concept to be represented formally, before discussing the details of its implementation. A similar point has been made by Daniel Peterson (2015) in distinguishing between intuitive and theory-relative accounts—whereas intuitive accounts rely on one’s temporal intuitions about time reversal to work out the form of the mathematical transformation, theory-relative accounts start off assuming that a particular theory is time-reversal invariant and from such an assumption they work out the form of the mathematical transformation that leaves the theory invariant. I think Peterson’s approach is on the right track, but I mean to take a step further: he does not overtly defend the existence of *metaphysical* underdetermination—his analysis is mainly based on epistemic (or theoretical) features (i.e., our intuitions about time reversal, or some overarching principle guiding theory construction), rather than on metaphysical aspects. My focus here is to dig a bit into those metaphysical aspects that drive our intuitions about how time reversal ought to be implemented. And this interestingly holds true for both intuitive and theory-relative accounts as I will show shortly.

The basic idea is then that the formal representation of time reversal is guided by some previous *conceptualization* of what time reversal is. For instance, a good deal of what time reversal conceptually is depends on what time conceptually is—after all, intuitively, time reversal is the reversion of time, but what is it meant by time? Prima facie, diverging notions of time can lead to different ways to understand what its reversion could amount to. In consequence, the representation of time reversal could be different. This does not mean that any conceptual understanding of time reversal and any implementation thereof is going to be equivalent, or equally valuable. It can well be the case that some conceptualizations of time reversal led to dead-end street, or to useless mathematical implementations. Be that as it may, it does not change the fact that the representation is secondary, meant to represent some concept previously adopted. The same holds for the concept of *invariance*. As was noted in passing, invariance basically means that some structure is preserved under a transformation (e.g., the space of solutions). However, invariances are not easy to come by in most physical theories. They only hold in very special cases and under extraordinary conditions.^4^ Despite this, the role of invariances is paramount, what is symptomatic that such special cases and extraordinary conditions are physically, and philosophically, relevant. But this assumption does not come for free and can also be challenged.

All these points would be relatively unproblematic if there were solid agreements on, for instance, what time is. However, this is not the case. One of the most pressing problems to understand the concept of time reversal is that, first, there is no consensus about what time is and, second, there is then no consensus about what reversing time might be. This is doubly problematic because our intuitions about what reversing time is have no solid ground to cling to. For instance, we could not know what space is, but we can in reality move things to left and right, to up and down; we also have mirrors, which simulate what inversing space might be. Time is of a radically different nature, since its inversion is not only unfeasible, but also hard to imagine. In one way or another, we must rely on some concept of what time is and then to rely on our intuitions about what its inversion might be. The most intuitive picture is that of thinking of time in terms of change, and therefore, thinking of reversing time in terms of reversing change. This concept of time reversal is particularly useful in physics, though, as I will show lately, is far from self-evident. Regardless these disputes, it seems clear to me that it would be an error to use the physical implementation of time reversal to clarify what time reversal is, since the former already supposes the latter.

In what follows, I present three cases in which, I believe, different conceptualizations of time reversal arise in different theoretical domains. I mean these different conceptualizations to feed the idea that time-reversal invariance suffers from some, at least moderate, metaphysical underdetermination. If these different conceptualizations exist, then it comes as no surprise to have disputes over alternative implementations within a physical theory. Although I will not defend any side in these quarrels, the message I want to convey is that disputes over implementations (or representations) are secondary; they stem from disputes over concepts. The first example centers in the notion of time reversal in non-relativistic quantum mechanics. In this case, the usual way to represent time reversal is contingent upon taking time reversal as motion reversal, which entails a relational view of time. My second example addresses the concept of time reversal and its formal implementation in classical electromagnetism. In concrete, the idea is that how time reversal is to be implemented in classical electromagnetism is contingent upon which the basic properties of the theory are, and how the temporal orientation is conceived in one’s ontology. Finally, my third example draws the attention towards the concept of time-reversal *invariance* in classical mechanics. I submit that whether time-reversal *invariance* holds in classical mechanics is contingent upon the ontology of classical mechanics, in particular, whether phenomenal or non-conservative forces are regarded as real or not.

## Time reversal in non-relativistic quantum mechanics

In non-relativistic quantum mechanics (NRQM henceforth), time reversal is represented by an anti-unitary operator that reverses the time coordinate and takes the complex conjugate over the quantum state. This means that the implementation of time reversal is given by an operator $\tau$ such that

$$\tau :t \to - t,$$

$$\tau :\psi \to \psi^{*} ,$$

$$\tau :X \to X,$$

$$\tau :P \to - P,$$

$$\tau :\sigma \to - \sigma ,$$

where $t$ represents the time coordinate, $\psi$ the wave function, $X$ the position operator, $P$ the momentum operator, and $\sigma$ spin. This implementation of time reversal leaves the Schrödinger equation invariant. Therefore, it is broadly accepted, the Schrödinger equation is time-reversal invariant.

Why time reversal must be represented in such a way has been extensively discussed in the literature. Almost any quantum–mechanical textbook justifies succinctly why this is the right representation of time reversal (see, for instance, Gasiorowicz, 1966; Messiah, 1966; Ballentine, 1998, among others). More thorough justifications have also been provided in the philosophical literature, even though they differ in the details and emphases (see Sachs, 1987; Roberts, 2017; Lopez, 2021). The reader is referred to the references therein for further details. What is interesting in the context of this article is that some have disagreed on this orthodox implementation, which has led to disagreements on whether the Schrödinger equation is time-reversal invariant or not.

Craig Callender (2000) has argued that NRQM is non-time-reversal invariant since the time-reversed Schrödinger equation is *not* equivalent to the original Schrödinger equation (Callender, 2000, p. 262). According to him, the time-reversal transformation only changes the sign of the time derivatives, which would amount to inverting the order of time. Callender’s view of time reversal is one in which the inversion of the direction of time is the temporal analogue of mirror handedness, that is, a reflection. Therefore, the *concept* of time reversal is that of a mirror symmetry, which is to be simply implemented by a transformation such that

$$\tau :t \to - t,$$

This, according to Callender, would be enough to formally represent the concept that time reversal—it is just a reflection on the temporal axis. The transformation of physical magnitudes (e.g., momentum, energy, velocity, etc.) under time reversal will ultimately depend on whether they logically supervene on switching the sign of the time coordinate (Callender, 2000, p. 254). The justification of why the Schrödinger equation is non-time-reversal invariant is that the representation of Callender’s conceptualization of time reversal invariance is one in which the magnitudes are transformed in such a way that the future-headed and past-headed quantum states are not time-reversal symmetric, that is, non-mirrored images.^5^ In his line, the *implementation* of time reversal is to be given by a unitary operator.

David Albert (2000) has also claimed that the common view of time reversal in NRQM is not right. For him, time-reversal invariance means that a physical theory entails that “whatever can happen can also happen backward”, or alternatively, “that the theory offers identical algorithms for inferring towards the future and the past” (2000, p. 11). What Albert has in mind is that any physical process can be viewed as some infinite sequence $S_{i} \ldots S_{f}$ of instantaneous states. Importantly, each instantaneous state is a complete and independent description of the state of the world at a certain time. If $S_{i} \ldots S_{f}$ is a process occurring forward, then $S_{f} \ldots S_{i}$ is just the sequence of a process occurring backward. Hence, there are two key concepts in Albert’s construal of time reversal: (a) it basically amounts to reversing the order of a sequence of instantaneous states, and (b) instantaneous states are complete and independent descriptions of the world at a single time.

Albert is not very clear about how such a concept of time reversal must be formally implemented in NRQM, but it is reasonable to suppose that, since each instantaneous state is genuinely independent and a complete description of the physical world, any set of properties necessary to provide such a complete description should not switch sign under time reversal (see Peterson, 2015 for discussion); if they switched sign because they are, for instance, a first time derivative (e.g., velocity) and they are part of the complete description of the world at a single time, then the states would no longer be genuinely independent. For this reason, NRQM turns out non-time-reversal invariant under Albert’s view—the sequence of complete descriptions of the quantum world is given by a series of quantum instantaneous states (e.g., wave functions $\psi \left( x \right)$), which should be left unchanged under time reversal. But, if this is so, then, $\tau :\psi \to \psi$, which amounts to implementing time reversal by a unitary operator. Therefore, the sequence $\psi_{i} \ldots \psi_{f}$ is not symmetric with respect to $\psi_{f} \ldots \psi_{i}$. To sum up, Albert’s conceptualization of time reversal is one in which an inversion of the direction of time is an inversion of a sequence of instantaneous states, where only basic and independent properties are involved. Its implementation is therefore to be given by a transformation that simply inverts the sequence but leaves the basic magnitudes untouched.^6^

More recently I have argued (2019, 2021) that whether NRQM is time-reversal invariant or not crucially depends on one’s concept of time. According to my view, the traditional implementation of time reversal is meant to represent an inversion of change (in particular, of motion), but an alternative conceptualization of time might lead to a different representation (2019, p. 10). The argument goes roughly as follows: the traditional view of time reversal requires some physical magnitudes to transform in a specific way (e.g., $\tau H\tau^{ - 1} = H$, $\tau P\tau^{ - 1} = - P$); but such transformations are required to represent the idea of inversion of the direction of change; an anti-unitary operator is the only that can successfully carry out such transformations. Therefore, if time reversal means change reversal, then the anti-unitary time-reversal transformation is the only adequate one. In Lopez (2021), I claim that this implementation of time reversal is contingent upon a philosophical background that provides the necessary conceptual tools to justify why time reversal must transform certain magnitudes in a specific way.

Though I defend the orthodoxy against Callender and Albert (Lopez 2021), I do open the door to deep, genuine disagreements. The philosophical background that underpins the orthodox implementation of time reversal is temporal relationalism. But if temporal relationalism is rejected, then the orthodox view “loses much of its persuasive force” (2021, p. 14,285). Hence, an alternative philosophical background can defend an alternative representation of time reversal. For instance, it could be suggested that Callender’s view on time reversal contains some substantivalist assumptions with respect to time, since Callender distinguishes between time reversal and “Wigner” reversal (or motion reversal). It would entail that motion reversal is a different concept to that of time reversal, which can be read as a rejection of temporal relationalism. Be that as it may, the same point still holds—divergencies over the implementation of time reversal are motivated by divergencies over its conceptualization. Since the implementation of time reversal now strongly resorts to a philosophical background, if there exist motivations to dispute the philosophical background, then time reversal would suffer from metaphysical underdetermination.

Let me square the scope of these claims. The point I want to make here is that there exists a genuine case of metaphysical underdetermination of time reversal because we can understand time differently. This does not imply that one’s view of time *determines* a specific concept and/or implementation of time reversal. To prove that there is underdetermination such a strong relation is not necessary, but it is enough to show that there is sufficient conceptual room for alternative conceptualizations and implementations. In the previous literature, I believe, this has not been so clear. Callender’s and Albert’s work do not show that there is underdetermination, but that people have been largely mistaken in implementing time reversal because they have been largely mistaken about what time reversal is. In my previous work, a thorough justification of the orthodox view is provided, but without showing clearly that there is a clear case of metaphysical underdetermination—after all, my defense of the orthodoxy relies on temporal relationalism, but I did not fully recognize that temporal relationalism requires an independent justification and that temporal substantivalism can make a good case against orthodox views [this is however explicitly mentioned in Lopez (2019)]. My claim here is that there is a genuine case of metaphysical underdetermination, if alternative ways to understand time (and time reversal) can lead legitimately to alternative implementations.

All of this emphasizes that whether time reversal is implemented by an anti-unitary or unitary operator is contingent upon what it is conceptually meant by time reversal and by time. In Callender’s view, time reversal conceptually means to perform a reflection upon the temporal axis, analogous to a mirror symmetry transformation. That is the basic transformation, upon which the transformations of physical magnitudes will supervene. There is nothing in the physics or mathematics of time reversal that conceptually promotes (or rejects) such a view. It may well be a physically uninteresting way to construe time reversal, since it might lead to no feasible applications; but Callender’s heterodox representation of time reversal is faithful to its conceptualization. Note that the debate fully moves now onto a philosophical terrain—is it right to conceptualize time reversal as a mere reflection? The same goes for Albert’s view, which resorts to viewing time reversal as an inversion of the order of instantaneous states (I will come back to Albert’s view in the case of time reversal in classical electromagnetism). In my approach, temporal relationalism plays a major role in justifying the orthodox view, but it also entails (though it is not explicitly mentioned) that there is ultimately some metaphysical underdetermination looming over the debate, to wit, whether temporal relationalism should be endorsed or not.

To come back to the Dynamical Argument, the metaphysical underdetermination of time reversal in NRQM requires us to qualify P2—it is not true that physical theories are time-reversal invariant simpliciter, but they are time-reversal invariant *if* time and time reversal are conceptualized in a specific way (e.g., Wigner Reversal, in Callender’s view; Temporal Relationalism, in my view; an inversion of non-instantaneous states, in Albert’s). This shows that the debate about what is time reversal has serious repercussions on subsidiary discussions. Employing my characterization for simplicity, the Dynamical Argument for the case of NRQM can be reformulated as follows:

NRQM-P1. If the dynamics of NRQM is time-reversal invariant, then a primitive direction of time is unwarranted.

NRQM-P2. It happens that NRQM is time-reversal invariant, *if temporal relationalism is adopted*.

NRQM-P3. Temporal relationalism is adopted.

NRQM-C. Therefore, a primitive direction of time is unwarranted in NRQM.

The argument is now correct, but it is worth highlighting that the discussion hinges now upon NRQM-P3. The problem in the end is not really about which is the right implementation of time reversal in NRQM, but about the reasons we have to adopt temporal relationalism (or Callender’s or Albert’s views). Once again, this opens the door not only to underdetermination, but also to heterodox views (e.g., Callender’s or Albert’s) against more traditional approaches to time reversal. But it is important to note that any defense or attack is primarily to be articulated philosophically, since it aims to flesh out the *concept* of time and time reversal.

## Time reversal in classical electromagnetism

In the case of NRQM, I have focused on how our conceptualization of time and time reversal influences its formal representation, leading to metaphysical underdeterminacy. A similar case arises in classical electromagnetism (CEM, henceforth), though it does not concern time, but the ontology of the theory.^7^ In particular, it concerns two ontological aspects. First, which properties are to be considered basic (or primitive) within CEM and whether they may switch sign under time reversal or not. Second, if a temporal orientation is something over and above the temporal direction in the series of CEM instantaneous states. I start by introducing the orthodox view, and then the disagreements.

According to textbooks, time reversal in CEM is to be implemented by a transformation $\tau$ such that

$$\tau :t \to - t$$

$$\tau :{\mathbf{B}} \to - {\mathbf{B}}$$

$$\tau :{\mathbf{E}} \to {\mathbf{E}}$$

$$\tau :{\mathbf{j}} \to - {\mathbf{j}}$$

$$\tau :{\mathbf{F}} \to {\mathbf{F}}$$

That is, time reversal switches the sign of the time coordinate *t* (as expected) and of the magnetic field **B** and the current **j**, while leaving the electric field **E** and the electro-magnetic force **F** unchanged. The rationale is the following. The transformation of the time coordinate is basic, so it needs no further justification. The magnetic field **B** can be expected to flip sign under time reversal because it should be treated as a velocity. If time reversal is meant to change the direction in which electrons flow in a wire (and we expect time reversal to change the sign of the velocities of electrons in a wire), then it would induce a sign change of the magnetic field (I will come back to this afterwards). The Lorentz force **F** is defined in terms of a particle’s charge (*q*) moving with a velocity (*v*) in an electric (**E**) and magnetic field **(B).** Since it can be simply viewed as ${\varvec{F}} = dp/dt$, it is expected to not change its sign. Similarly, the electric field (defined in function of **F** and the charge *q*) should remain unchanged under time reversal. The current **j** is a first-time derivative, then it is expected to change sign.

The textbook implementation of time reversal leaves CEM invariant—Maxwell’s equations and the Lorentz Force Law preserve their space of solutions if time reversal is represented by $\tau$. Yet, Albert has argued against this conclusion on the basis of *his* definition of time reversal. In the special case of CEM, what is relevant according to his conceptualization of time reversal is the properties that are required to give a complete and independent description of CEM states at a single time. This entails basic properties to be carefully distinguished from derivative properties. If a property is regarded as basic (or primitive), then it must not switch sign under time reversal (otherwise, the state would be rendered dependent). In Albert’s view of CEM, the magnetic field **B** *is* a basic property since it enters the theory as an ingredient in the complete description of an instantaneous state, and not as a dynamical condition dependent on past or future states. Therefore, it must not switch sign under time reversal, $\tau :{\mathbf{B}} \to {\mathbf{B}}$**.** The argument is straightforward: time reversal must only switch the sign of the derivative properties and keep the sign of the basic properties (Albert’s assumption on time reversal). The magnetic field ${\mathbf{B}}$ is a basic property (Albert’s assumption on the ontology of CEM). Therefore, it must not switch sign under time reversal.

Albert’s view of the time-reversal transformation in CEM is unusual and has received many criticisms on different grounds (notably, Earman, 2002; Malament, 2004; North, 2008; Arntzenius & Greaves, 2009; Struyve, 2022). The discussion is extensive and subtle in many respects, so the reader is referred to the references for further details. For instance, Arntzenius and Greaves (2009) have objected Albert’s view on the basis of simplicity—Albert’s view of time reversal and his commitment to a Newtonian spatial–temporal structure is less parsimonious than standard approaches. If it is advisable to be committed to more parsimonious structures, then Albert’s view must be rejected (2009, p. 13).

What I would like to remark here is another potential source of divergences, related to the basic ontology that Albert proposes. Albert defends an active time-reversal transformation upon the physical states that presupposes a distinction between basic and non-basic properties, since time reversal must act upon magnitudes in a specific manner depending on whether they are basic or not. To resist Albert’s representation of time reversal, his conceptualization of time reversal and CEM can be rejected. One of the lines of attack is to argue that the magnetic field is not actually a basic property, but a derivative one. A line of argumentation against Albert can run as follows: Magnetic fields are produced by electric currents (e.g., microscopic currents associated with electrons), which are the rates of the flow of electric charges. Since it is reasonable to expect time reversal to change the direction of the electric charge flow, it is also reasonable to expect time reversal to induce a change in the sign of the magnetic field. But note that the discussion hinges no longer on time reversal, but on how magnetic fields should be understood. One may have different reasons for adopting either view (this one may be more adequate in a physical explanation), but it is worth noting that Albert’s approach to time reversal forces the discussion to center in the ontological premise about whether the magnetic field must be regarded as basic or not, regardless of whether it is useful or not to adopt his view. To put it differently, Albert forces to move the discussion from time reversal to which is the fundamental ontology of the theory.

David Malament (2004) has also replied to Albert’s view, but on different grounds. He does not directly concern whether the magnetic field is a basic property or not; actually, he thinks that whether time reversal changes the sign of the magnetic field is a “distraction” (Malament, 2004, p. 296). He rather proposes an alternative view of time reversal, one in which the transformation is a *geometric* transformation. After all, as Jill North puts it, the most natural way to construe time reversal is as a transformation that just flips the temporal orientation itself (2008, p. 212). The most straightforward implementation of time reversal is hence one that represents the flipping of the temporal orientation as an inversion of the space–time structure, without taking care of shifting particles and field values. It is worth remarking that Malament’s geometric transformation is not merely a change to a passive view of time reversal. That would render the view uninteresting. The transformation of the temporal orientation is meant to be more substantive than merely a change in the description of the CEM laws.

In Malament’s view, the geometric time-reversal transformation keeps fundamental quantities unaltered, but it changes how they are disposed with respect to a given temporal orientation. In this way, time reversal will change the fields and fundamental properties on a space–time manifold as determined relative to one temporal orientation *to* the corresponding fields and fundamental properties on a space–time manifold as determined relative to other temporal orientation (2004, p. 306). But these changes are produced by flipping the temporal orientation, not because time reversal acts directly upon them. Malament’s view, then, rejects Albert’s premise that time reversal only acts upon the basic properties that provides a complete description of instantaneous states. He alternatively defends the view that time reversal acts mainly upon the temporal orientation.

To put all the pieces together. The answer to whether classical electromagnetism is time-reversal invariant or not follows from how time reversal is represented; in particular, whether it switches the sign of the magnetic field or not.^8^ But this representation is contingent upon the ontology of the theory in two senses. First, it is contingent upon whether the magnetic field is a basic property or not. For Albert, it is, but it could be argued that it is not, as is indeed usually understood. Once again, I am not concerned with who’s right on this. My point is that alternative conceptualizations of time reversal (i.e., upon which we think time reversal ought to act) may diverge and, with it, their representations. To put it differently, Albert provides just the right sort of representation for *his* conceptualization of time reversal. Alternative views disagree first and foremost on Albert’s conceptualization.

Second, the representation is contingent upon whether time reversal is meant to primordially flip the temporal orientation. This point is complex and can be disentangled in different theses, but I want to draw the attention to one of Malament’s assumptions: there *is* a temporal orientation over and above any sequence of CEM states. In Albert’s concept of time reversal, the reversion of time is given by inverting the order of a sequence of states. In fact, the direction of the sequence is defined independently of an external temporal orientation, as it were. When North, in spelling Malament’s view out, says that it is natural for time reversal to transform the temporal orientation itself, it implies that the temporal orientation is *something* over and above the intrinsic direction of any sequence of states. The ontology then is substantively different. Basic properties and field coexist along with the temporal orientation in Malament. In Albert, it is not clear that there be a temporal orientation independently from the temporal order of the series of states.

On this basis, two additional premises can be added to the Dynamical Argument for the case of CEM:

CEM-P1. If the dynamics of CEM is time-reversal invariant, then a primitive direction of time is unwarranted.

CEM-P2a. It happens that CEM is time-reversal invariant, *if the magnetic field is non-basic* (the negation of Albert’s view), or

CEM-P2b. It happens that CEM is time-reversal invariant, *if a temporal orientation exists over and above the sequence of states* (Malament’s view)

CEM-P3a. *The magnetic field is non-basic*, or

CEM-P3b. *A temporal orientation exists*

CEM-C. Therefore, a primitive direction of time is unwarranted in CEM.

There may be good reasons to uphold CEM-P3a and/or CEM-P3b. However, my central thesis still stands. There is no obvious and metaphysically independent way to support a determined conceptualization of time reversal; then, there is no obvious and metaphysically independent way to support a determined *representation* of time reversal. Both premises should be argued for on a philosophical basis.

## Time-reversal invariance in classical mechanics

In the two previous cases, metaphysical underdetermination emerged in assessing the concept of time reversal in the light of the nature of time and a theory’s ontology. I now slightly change the focus to the concept of time-reversal *invariance*. The Dynamical Argument as formulated in Sect. 1 states that time-reversal *invariance* is the relevant feature of physical theories to elucidate whether the world comes fundamentally equipped or not with a direction of time according to such theories. But what does ground the claim that a physical theory is time-reversal *invariant*? Or a bit differently, under which conditions is a theory accepted as genuinely time-reversal invariant?

These questions are valid in any theoretical framework (quantum and non-quantum, relativistic and non-relativistic), but I circumscribe myself here to Newtonian classical mechanics (NCM, henceforth) for simplicity’s sake. The conventional wisdom says that time reversal is a transformation such that

$$\tau :t \to - t$$

$$\tau :x \to x$$

$$\tau :v \to - v$$

where $t$ is the time coordinate, $x$ represents positions, and $v$ velocities. Under such a transformation, Newton’s laws turn out time-reversal invariant in the plain sense that their space of solutions is preserved in applying such transformations. That is, a Newtonian evolution is transformed into a *dynamically possible* Newtonian evolution under such a representation of time reversal. The conclusion, via the Dynamical Argument for this case, would be that there is not primitive direction of time in NCM to the same extent that there are, for instance, not absolute positions.

Keith Hutchison (1993, 1995) challenged the conventional wisdom on time-reversal invariance in NCM. He argued that the very formulation of time-reversal invariance for physical laws is problematic, but not because of the notion of time reversal, but because of the narrow and exclusive reading of the concept of “law” in such a formulation (1993, p. 315). Hutchison’s view is that the conventional wisdom just ignores a massive number of scientific regularities that are not time-reversal invariant, privileging a special group that it is. Why this is so, Hutchison holds, is vague. He argues that is not clear why, for instance, a crude empirical approximation does not qualify as a law, when it is of common use in physical practice. Even more drastically, he claims that “it is inappropriate to force the laws of NCM into either the reversible [time-reversal invariant] or the irreversible [non-time-reversal invariant] category” (1993, p. 316),^9^ since the forces are ultimately which decide whether an equation is time-reversal invariant or not. The crucial point is that the overwhelming majority of such scientific regularities (which involve different kinds of forces) are non-time-reversal invariant. Hence, on which grounds is NCM said to be time-reversal invariant as a whole? Under this revised notion of time-reversal invariance for the laws of classical mechanics, the claim is vague, if not misleading.

Hutchison’s argument ultimately relies on what he views as an unwarranted division in one’s ontology to decide whether a theory is time-reversal invariant or not—fundamental laws, on the one side, and phenomenological laws, on the other. The claim that NCM is time-reversal invariant unjustifiably privileges highly idealized laws (or, better, conservative forces), whereas rejects phenomenological laws (or, better, non-conservative forces) as ontologically relevant. Note that Hutchison’s view is already enough to qualify the Dynamical Argument, regardless of whether there are good and sound reasons to draw such a distinction—P1 in the argument as presented in Sect. 1 should explicitly state that *fundamental* laws (understood as those laws that only involve conservative, fundamental forces) are the relevant to assess time-reversal invariance in the context of the Dynamical Argument.

But what is meant by “fundamental” laws? Is this division warranted? In a reply to Hutchison, Craig Callender says: “when asking if the universe is TRI [time-reversal invariant], we desire to know whether it is *at bottom* time-reversal invariant. We make an ontological assumption”, which is the following: “there are really only particles in motion and inter-particulate (distance-dependent) forces. That is to say, as Feynman does, ‘there are no non-conservative forces!’” (1995, p. 333). So, according to Callender, an ontological assumption about what is the fundamental ontology of NCM draws the line between fundamental and phenomenal forces. Then, in assessing time-reversal invariance an additional assumption is made—we are not interested in testing time reversal on *any* Newtonian regularity, but only on those in which conservative, fundamental forces intervene. It follows from this that “the crude empirical approximations”, as Hutchison puts it, that physicists have so well mastered and employed daily are not of philosophical interest to assess time-reversal invariance.

I believe that Callender’s argument is good. It indeed does a great job in justifying why it is so common to think of NCM as time-reversal invariant, despite the numerous forces that violate it. Nonetheless, as was previously mentioned, the Dynamical Argument needs some amendments to work as intended:

NCM-P1. If the *fundamental* dynamics of NCM is time-reversal invariant, then a primitive direction of time is unwarranted.

NCM-P2. It happens that the *fundamental* dynamics of NCM is time-reversal invariant.

NCM-C. Therefore, a primitive direction of time is unwarranted in NCM

It is worth noting that if the qualification is not introduced, then Hutchison is absolutely right in pointing out that there is some vagueness in the statement of time-reversal invariance, since there would be obvious cases where time-reversal invariance is violated. It does not mean, however, that the qualification is enough. In a 1995 article, Hutchison claims that such fundamental laws are generally idealizations, so it should not come as a surprise that the time-reversal invariance they “predict is not witnessed in the world about us, because the world about us is enormously different to the world presumed” in such idealizations (Hutchison, 1995, p. 232). It seems to me that Hutchison is pointing to a hidden assumption in Callender’s argument—why should we take idealizations metaphysically seriously to tailor one’s ontology? He is not explicit on this, but if my reading is adequate, then he is also referring to an old problem in philosophy of science—are idealizations truth-conducive? Whether we accept that NCM dynamics is time reversal invariant or not depends on accepting that only conservative forces exist (at least at a fundamental level). Now, in order to accept such an assumption, further arguments are required—why should idealizations guide one’s ontology?

To put it differently, it is clear that the Dynamical Argument seeks to uncover some fundamental feature about world’s temporality. However, the qualification just introduced centers in, e.g., idealized forces; or, differently, in idealized laws that have been stripped off the features of our world (e.g., non-conservative forces). So, are they reliable sources for ontology? More importantly, do they affect the reliability of the concept of time reversal? After all, it is assumed that the concept of time reversal is somehow connected to the world’s temporal structure; but if the concept of time reversal is only valid (or is only instantiated) in highly idealized contexts, where only conservative forces exist, then it seems we are forced to adopt all of this too. I do not want to get into details here, but it is enough to point out that if some sort of realist credibility is not given to idealizations, it is not easy to block Hutchison’s refusal to take ontologically seriously the distinction between fundamental and phenomenal forces or laws. Be that as it may, this is another source of metaphysical underdeterminacy at the heart of time-reversal invariance, which is relative to how we are to conceive of a theory’s fundamental ontology, whether highly idealized models will be regarded as privileged or not, etc.

## The dynamical argument revisited

In the previous sections, I have presented three cases in the philosophy of physics literature where time-reversal invariance seems to suffer from some form of metaphysical underdetermination. In one way or another, the three cases analyzed call for qualification and completion of the Dynamical Argument, if it is meant to work as intended. On the one hand, I believe that they help underpin the argument in order to avoid vagueness or argumentative gaps. The Dynamical Argument, in the general and unqualified fashion of Sect. 1, is not good enough. Any of the refined, qualified versions, to my mind, are improvements that can be of philosophical interest.

On the other hand, they however open some flanks of attack. In the case of NRQM (Sect. 2), temporal relationalism is an assumption that can be rejected on philosophical grounds, eroding orthodox views on time reversal in NRQM. In CEM (Sect. 3), some disputable ontological assumptions have to be adopted, if time-reversal invariance is to hold. Finally, in NCM (Sect. 4), a distinction between fundamental and phenomenal laws needs to be drawn, and with it, an ontology of purely conservative forces posited. To generalize these theses, we can say that the metaphysical underdetermination of the concept of time-reversal invariance manifests itself in

- Which is the concept of time involved (e.g., temporal relationalism, temporal substantivalism)
- Upon which time reversal is to act (e.g., upon instantaneous states, upon the temporal orientation)
- Of which time-reversal invariance is to be predicated (e.g., fundamental laws, phenomenal laws)

As was mentioned in the Introduction and Sect. 1, these bullets points affect the *concept* of time reversal. That presupposes that different stances on these points disagree on the conceptual content of what is meant by time reversal. Their *representations* in the mathematical apparatus of a physical theory will seek to capture and model the most salient features of each conceptualization. It does not come as a surprise then that if time reversal is susceptible to different conceptualizations, there may be alternative representations. After all, metaphysical underdeterminacy means just that.

As was mentioned many times before, this does not mean that any conceptualization is equally valid, useful, or philosophically appropriate. We can indeed have well-grounded reasons to defend the conventional understanding of time reversal in the different theories, and to also support all these assumptions and make the Dynamical Argument work as intended. Nonetheless, it does not circumvent the fact that the metaphysical underdetermination calls for further philosophical work. The first step, I believe, is to state the assumptions clearly and explicitly; then to argue for them on philosophical grounds.

## Data Availability

Not applicable.

## Footnotes

1 There are two broad views on the direction of time in physics –primitivism and non-primitivism. On the one hand, primitivism about the direction of time holds that time is fundamentally (irreducibly) directed, whatever we mean by ‘time’. One of the ways to conceive of this view is to regard the direction of time as being *built-into* the laws of physics (see Maudlin, 2002; Loewer, 2012). On the other hand, non-primitivism defends a reductive approach to the direction of time, where any temporally directed process *emerges from,* or *supervenes upon*, a temporally directionless basis. Non-time-reversal invariant laws would support primitivism, whereas time-reversal invariant laws would argue in favor of non-primitivism.

2 A good argument for supporting this premise runs as follows. Desirably, dynamical symmetries of a theory should match its space–time symmetries (see Earman, 1989, p. 46; North, 2008, p. 202). That is, symmetries found at the level of the dynamics of a theory are symmetries of the space–time structure that supports it. If the direction of time is to be found in the underlying space–time structure, a straightforward way to know it is by looking into the symmetries of the dynamics. See Horwich (1987) for a defense of time-reversal invariance as a nomological property.

3 Some clarification is in order. By ‘physical’ transformation I do not simply refer to an empirical, feasible transformation. The ‘physical’ transformation could be ideally conceived. Since we cannot in fact reverse time empirically, we rely on some idealizations to imagine how the world (and the laws of nature) would behave if time were reversed. To assess such a contrafactual within physics, we should grant that it is a physical transformation, regardless how idealized it may be.

4 See Belot (2013). The idea can naturally be upheld if a view similar to that of Nancy Cartwright (1983) is applied not only to laws, but also to symmetries. In general, symmetries are properties of the fundamental laws (what Cartwright calls ‘covering laws’). But if such laws, according to Cartwright, are only valid under very special conditions, then their symmetries will be also valid under very special conditions.

5 In particular, the Hamiltonian and the momentum operator do not transform in the adequate way to leave the dynamics of NRQM (as least in its unitary part) invariant. For further details, the reader is referred to the cited references.

6 Even though Albert is not very explicit about it, it can be argued that since the quantum state is *fundamental*, it gives a *complete* description of the state of the (quantum) world at a single time, and time reversal only flips signs of non-fundamental and non-autonomous magnitudes, there is no reason why the quantum state should be affected by time reversal. This probably goes hand-in-hand with his wave-function realism.

7 The relation between time reversal and ontology has also been explored in non-relativistic quantum mechanics (Allori, 2019). Valia Allori, for instance, criticizes that time-reversal invariance can be employed to shed light on the nature of time. She argues that some arguments concluding that non-relativistic quantum mechanics is non-time-reversal invariant crucially rely on the assumption that the wave-function represents a *physical* scalar field in configuration space (in deterministic frameworks). However, the relation between time reversal and ontology I want to stress slightly differs from that of Allori. My interest is to show that the very *definition* of time reversal in terms of the actions required to physically and formally represent an inversion of time can be different depending on which properties are taken as basic and which as non-basic.

8 In a recent paper, Ward Struyve (2022) has also stressed the relation between ontology and time reversal in classical electromagnetism. His argument is that, by changing the ontology of the theory, it can be made time-reversal invariant under the same conception of time reversal (Struyve, 2022, p. 17). Struyve hence shows that, under a proper choice of ontology, one can vary the notion of time reversal and still keep the theory invariant. Therefore, his argument is that the metaphysical underdeterminacy of physical theories (e.g., classical electromagnetism or non-relativistic quantum mechanics) allows us to keep a theory time-reversal invariant. I fully agree with Struyve’s conclusion. However, the point I want to make in this paper is slightly different, though complementary. Classical electromagnetism shows us that it is the very representation of time reversal which may suffer from metaphysical underdetermination. But if this is so, then classical electromagnetism is not time-reversal invariant simpliciter, but it requires further assumptions. The point is that these assumptions tailor the representation of the time-reversal transformation. So, in my case, I am not so much interested in showing that a theory can be rendered time-reversal invariant by changing the ontology, but in showing that physical theories are not time-reversal invariant simpliciter but require further assumptions.

9 It is worth mentioning that Hutchison seems to conflate time-reversal invariance with reversibility—while the former is a property of dynamical equations, the latter is a property of their solutions. See Castagnino and Lombardi (2009) for clarification.
